# Associations between negative symptoms and resting-state functional connectivity within social brain networks among individuals with early psychosis

**DOI:** 10.1038/s41537-026-00756-9

**Published:** 2026-04-17

**Authors:** Anna R. Knippenberg, Lawrence H. Sweet, Lauren Luther, Somin Kim, Gregory P. Strauss

**Affiliations:** 1 Department of Psychology, University of Georgia, Athens, GA USA; 2 Department of Psychology, University of Alabama at Birmingham, Birmingham, AL USA

**Keywords:** Human behaviour, Neuroscience

## Abstract

Negative symptoms of psychotic disorders are best represented within a hierarchical structure comprising two broad dimensions—Motivation and Pleasure (MAP) and Diminished Expressivity (EXP)—and five lower-level domains. The validity of these two dimensions and five domains is supported by associations with cognitive, psychological, and clinical outcomes. However, few studies have examined whether they are differentiated by distinct neural mechanisms. The current study examined the specificity of associations between the two dimensions and five domains and resting-state functional connectivity (RS-FC) within five large-scale brain networks critical for social behavior. Participants included 125 early psychosis (EP) patients and 58 healthy controls (CN) from the Human Connectome Project-Early Psychosis who completed resting-state functional magnetic resonance imaging (rsfMRI) scans. RS-FC was quantified in five social brain networks: affiliation network, aversion network, perception network, mentalizing network, and mirror network. Early psychosis patients exhibited significantly reduced RS-FC in social brain networks compared to CN, but no specific network was responsible for this effect. Reduced RS-FC in the mirror network was significantly associated with greater asociality, anhedonia, and avolition, while reduced RS-FC in the mirror and mentalizing networks was associated with more severe blunted affect. Findings suggest that some RS-FC networks align with the broader higher-order dimensions, while others align with the lower-level domains. Overall, the pattern of findings suggests that abnormal patterns of resting-state social brain network activation are broadly associated with the MAP dimension and not more selectively related to anhedonia, avolition, or asociality. Thus, findings suggest that the neurobiology of negative symptoms in EP is best captured at the level of the broader higher-order dimensions than the specific lower-level domains that make them up.

## Introduction

Psychotic disorders (PDs) are a leading cause of functional disability that is linked to high public health costs^1,2^. Negative symptoms—avolition, asociality, anhedonia, blunted affect, alogia—are a strong predictor of functional impairment^3–8^. Unfortunately, available pharmacological and psychosocial treatments have proven minimally effective at remediating negative symptoms^9–11^. Thus, these symptoms remain a critical unmet need in PD therapeutics.

Limited progress in treatment may stem in part from challenges in conceptualizing negative symptoms and their underlying mechanisms^12^. The DSM-5-TR defines negative symptoms as broad dimensions: Volition and Diminished Expression. Exploratory factor analyses (EFAs) of clinical rating scales consistently support the higher-order factor structure (emotional expressivity [EXP], motivation and pleasure [MAP]) that the DSM-5 conceptualization was based on refs. ^13–15^. However, more recent confirmatory factor analyses (CFAs) testing competing models of the latent structure of negative symptoms have cast doubt on the validity of the higher-order dimensions, which tend to have poor fit in CFAs. Instead, lower-level domains (anhedonia, avolition, asociality, alogia, blunted affect) and hierarchical models including both higher-order dimensions and lower-level domains have an excellent fit^16–21^. These CFA findings appear robust, having been replicated across the most contemporary clinical interview scales (Clinical Assessment Interview for Negative Symptoms [CAINS], Brief Negative Symptom Scale [BNSS], Scale for the Assessment of Negative Symptoms [SANS]), self-report questionnaires (Self-Evaluation of Negative Symptoms [SNS], Community Assessment of Psychic Experiences [CAPE]), all illness phases (clinical high risk, early psychosis, chronic), both sexes, numerous cultures/languages, and various mathematical approaches (e.g., CFA, network analysis)^22–25^.

To develop targeted treatments, empirical studies examining the neural processes associated with the lower-level domains are needed to determine whether each factor demonstrates unique mechanisms that are masked by the broader higher-order dimensions. Resting-state functional connectivity (RS-FC), delineating the magnitude of covariation in activity among brain regions across time, represents a powerful tool for examining how brain regions interact with one another^26^.

RS-FC fMRI studies have shown functional dysconnectivity across illness phases in PD^27–43^. In early psychosis (EP), evidence exists for both hyper- and hypoconnectivity in EP compared to CN^35–43^. While both hyper- and hypoconnectivity have been associated with greater negative symptoms in chronic PD^44–49^, hypoconnectivity largely correlates with greater negative symptoms^39,50^ and functioning^35,51^ in EP.

Individuals with EP have been shown to exhibit hypoconnectivity within the default mode network (DMN), interconnected brain regions that exhibit greater activity when at rest compared to performing tasks^36–39^, as well as other brain networks^35,40–42^ compared to CN. Hyperconnectivity in EP compared to CN has been primarily found within other thalamocortical^43^ and frontostriatal circuits^42^. Greater negative symptom severity has been associated with both hyper- and hypoconnectivity in EP^39,50,52^. Lute et al. found that hypoconnectivity within DMN, including synchrony between the medial prefrontal cortex and anterior temporal DMN subnetworks, was associated with more severe negative symptoms^50^. Cao et al. found that cerebello-thalamo-cortical hyperconnectivity predicted poor long-term reduction in negative symptoms in EP^52^.

A model of social brain network function proposed by Bickart et al. may be particularly pertinent given the involvement of social processes in the negative symptom domains that comprise the MAP dimension (e.g., detecting, processing, approaching, and avoiding social stimuli)^53^. Bickart et al. delineated five brain circuits relevant to social functioning in healthy individuals: the affiliation network, aversion network, perception network, mentalizing network, and mirror network (see Table 1 for regions of interest included in each network)^53^.

**Table 1:** Regions of interest in five large-scale social brain networks.

Social brain networks	Regions of interest (ROI)						
Affiliation	dTP = dorso medial temporal pole	rACC = rostral anterior cingulate cortex	sgACC = subgenual anterior cingulate cortex	vmPFC = ventromedial prefrontal cortex	Ent = entorhinal cortex	PHip = para hippocampal cortex	vmSt = ventromedial striatum
Aversion	cACC = caudal anterior cingulate cortex	Ins = insula	SII = somatosensory operculum	vlSt = ventro lateral striatum			
Perception	lOFC = lateral orbito frontal cortex	vTP = ventro lateral temporal pole	FG = fusiform gyrus	STS = superior temporal sulcus			
Mentalizing	dmPFC = dorso medial prefrontal cortex	PCC = posterior cingulate cortex	Precun = precuneus	AngG = angular gyrus (temporoparietal junction)			
Mirror	pSTS = posterior superior temporal sulcus	IPS = intraparietal sulcus	PreMC = premotor cortex				

The affiliation network contributes to forming social bonds and prosocial behaviors^53^. In CN, increased RS-FC between regions of this network (e.g., ventral medial prefrontal cortex to amygdala) is associated with increased emotion regulation, essential for the approach and maintenance of social interactions^54,55^. Dysfunction in this network observed across various neurodegenerative diseases suggests that it can lead to emotional detachment and social withdrawal^56^. In PD, aberrant activation and decreased RS-FC in affiliation network regions (e.g., vmPFC) are associated with emotion dysregulation and difficulty resolving emotional conflict^57–59^.

The aversion network facilitates the integration of social information that prompts avoidance of harmful social situations. In CN, aversion network activation is linked to feelings of disgust and anger in social contexts^60,61^, whereas dysfunction is associated with impaired trust^62^. In PD, decreased activation in aversion network regions (e.g., insula, anterior cingulate cortex, somatosensory cortex) is associated with dysfunction in integrating internal states with external social cues and altered reactivity to aversive stimuli^63,64^.

The perception network detects and processes social stimuli, which is important for engagement in social interactions. Orchestrated by the amygdala, the perception network interacts with the salience network for rapid detection of salient stimuli^53,65^. In both CN and PD, activation within perception network regions is associated with processing emotional facial expressions, though these regions show decreased volume and structural abnormalities in PD^66–68^.

The mirror network mediates interpreting the intentions and beliefs of oneself and others^69^. In CN, the mirror network is associated with mentally simulating and interpreting others’ behaviors and emotions using sensory and motor processing^70^. In PD, decreased activation in mirror network regions (e.g., inferior frontal and ventral premotor cortex) when attributing positive behaviors is associated with a lack of inner simulation and empathy, as well as challenges with social experience sharing and imitation^71,72^.

The mentalizing network, which largely overlaps with the DMN, has been studied more extensively than the other networks^72^. In CN, increased RS-FC within the mentalizing/DMN network relates to greater feelings of loneliness^73–77^. In PD, increased activation in mentalizing network regions (e.g., precuneus/posterior cingulate cortex) is linked to negative biases in interpreting social information^72^.

These five circuits are critical for social functioning in healthy individuals. The social processes that they govern have a high degree of overlap with the behavioral deficits involved with MAP negative symptoms. For example, the affiliation network overlaps with the internal experience social motivation component of asociality, whereas the aversion network overlaps with the social withdrawal component of behavioral asociality. The perception, mirror, and mentalizing networks relate to aspects of social cognition that are critical for initiating social activities (i.e., the behavioral component of asociality).

The current study evaluated the specificity of the association between the lower-level MAP negative symptom domains and RS-FC within the five brain networks important for social functioning. Given that social functioning is key to asociality, it was hypothesized that decreased RS-FC within each of the social brain networks would be associated with more severe asociality in EP, and this association would be significantly stronger than the other two MAP domains (hypothesis 1). It was also hypothesized that individuals with EP would exhibit significantly lower RS-FC compared to CN in each network (hypothesis 2).

## Methods

### Participants

Data were collected from 125 individuals with early phase psychosis (i.e., within 5 years of initial psychotic symptom onset) (EP) and 58 healthy controls (CN) from the Human Connectome Project-Early Psychosis Disease Project (HCP-EP; Table 2)^78–81^. EP included schizophrenia, schizophreniform, schizoaffective, psychosis not otherwise specified, delusional disorder, or brief psychotic disorder (non-affective psychosis; *N* = 34), as well as major depression with psychosis or bipolar disorder with psychosis (affective psychosis; *N* = 91) (see ref.^81^ for Inclusion/Exclusion criteria). Data were analyzed from the HCP-EP Release 1.1, which was the most updated data release at the time of this study.

**Table 2:** Demographic characteristics of the HCP-EP analyzed sample.

	EP = 114 *M (SD)*	CN = 46 *M (SD)*	Test statistic, *p*
Age	23.4 (3.36)	22.8 (3.49)	*F* = 0.91, *p* = 0.36
% Female	36.84%	34.78%	*x*^*2*^(1) = 0.004, *p* = 0.95
Race			*x*^*2*^(4) = 33.71, *p* < 0.001
White	67.5%	30.4%	
Black or African American	15.8%	60.9%	
Asian	11.4%	4.4%	
Multiracial	0.9%	2.2%	
Unknown or not reported	4.4%	2.2%	
Ethnicity			*x*^*2*^(2) = 1.30, *p* = 0.52
Hispanic or Latino	7.9%	6.5%	
Not Hispanic or Latino	89.5%	91.3%	
Unknown or not reported	2.6%	0.0%	

Education level was not reported.

*EP* early psychosis, *CN* controls.

Of the initial HCP-EP sample (EP = 125; CN = 58), MRI preprocessing quality control and exclusion due to high motion yielded the final analyzed sample of 114 EP and 46 CN. The processed and analyzed samples showed no significant differences in resting-state connectivity or clinical characteristics (Supplementary Table 1). The analyzed EP exhibited mild to moderate negative symptoms, typical for early psychosis^82,83^. EP and CN did not significantly differ on age, sex, or ethnicity.

### Procedures

Participants completed clinical symptom ratings and resting-state magnetic resonance imaging (rsMRI). RS-FC analyses evaluated the synchrony between ROIs in five social brain networks – aversion, affiliative, perception, mentalizing, and mirror networks – that were delineated by Bickart et al. as important for social functioning in healthy individuals^53^. All measures were administered by staff trained by clinical psychologists and psychiatrists with expertise in clinical and cognitive assessments^84^. The Structured Clinical Interview – Research Version (SCID-5-RV)^85^ was used to assess EP and CN participants to rule out non-psychotic EP or psychosis related to substance abuse or organic disease. It was also used to confirm that CN participants did not meet criteria for bipolar disorders, major depressive disorder, or schizophrenia. Negative symptoms were evaluated with the Clinical Assessment Interview for Negative Symptoms (CAINS)^14^. The Motivation and Pleasure (MAP) scale is the average of items 1–9. Anhedonia is the average of items 3, 4, 6, 8, and 9. Asociality is the average of items 5 and 7. Avolition is the average of items 1, 2, 5, and 7. The Expression (EXP) scale was calculated for exploratory analyses and is the average of items 10–13. Alogia is the average of items 10 and 11. Blunted affect is the average of items 12 and 13. Correlations between CAINS domains are reported in Supplementary Table 2.

### MRI acquisition

Scans were conducted using three Siemens MAGNETOM Prisma 3T scanners at Brigham and Women’s Hospital (BWH), McLean Hospital, and Indiana University (IU). BWH and IU utilized a 32-channel head coil, while McLean used a 64-channel head & neck coil (with neck channels off). The scan sequences included T1-weighted (MPRAGE) and T2-weighted (SPACE) structural scans at 0.8 mm isotropic resolution, and four 5-min and 46 s rsfMRI acquisitions (approximately 23 min) at 2 mm isotropic resolution with a multiband acceleration factor of 8, TR of 720 ms, and two runs each with AP and PA phase encoding. The four resting-state sessions per participant were combined and treated as a single longer session in the analyses, given that they were collected during a single scanning session^84,86^. Total scanning time was approximately 65 min. Subjects were advised to remain still, with foam cushioning for head stabilization and noise-attenuating headphones provided. Real-time image reconstruction enabled quality assurance, allowing for rescanning if issues were detected (see ref.^81^ for additional MRI data acquisition details).

### MRI processing and analysis

Data were processed using AFNI (http://afni.nimh.nih.gov/afni/; RRID:SCR_005927). An AFNI proc.py pipeline was used to process the unprocessed HCP-EP data, including individual voxel-wise general linear modeling (GLM) to remove known sources of variance^87^. This pipeline included deobliquing, slice-time correction, volume outlier and excessive motion censoring, registration and alignment of echo-planar imaging (EPI) data to T1 anatomy, and application of a 3-dimensional 4 mm FWHM Gaussian blur. Quality control included checking for EPI-T1 misalignment and exclusion of participants with less than 9 min of valid data after censoring outlier volumes (>5% of volume voxels exhibit outlier values) and excessive movement (>0.20 mm observed inter-volume movement in any direction). A threshold of at least 9 min of analyzable resting-state data was used, as this duration balances reliability with sample size and external validity^88^.

Resting-state functional synchrony was calculated between each voxel of the brain and the mean time course of each of the seed regions (Table 1), producing one whole-brain seed map of Pearson’s *r*-values per seed region of interest (ROI). Each ROI served as a seed region once for each network. These maps were transformed to z-scores and used in two ways: (1) averaging voxels within each target ROI of each network to create mean seed-target effects for hypothesis testing and (2) characterizing voxel-wise whole-brain relationships to each seed ROI for comparison to prior literature.

For hypothesis testing, mean z-values were averaged across the mean effects of each target ROI of each network. A weighted average was calculated for each RS-FC brain network at the participant level such that the mean effects of each target ROI were weighted by its size relative to the network. Weighted ROI averages were summed to calculate weighted network averages. Weighted network averages were used as a within-network correction to account for the varying sizes of the ROIs within each network^89^. Bivariate correlations were used to quantify associations between each brain network synchrony (mean z-scores) and negative symptom scores.

### Data analysis

Analyses were performed on the analyzed sample (EP = 114; CN = 46). Bivariate correlations tested the primary hypothesized pattern of associations between five social brain networks and three motivation/pleasure (MAP) negative symptom domains via the CAINS (i.e., asociality, avolition, anhedonia). The Benjamini and Hochberg correction for multiple comparisons was applied to the primary analyses. The false discovery rate was set at *q* = 0.05. For each analytic family, *p-*values were ranked in ascending order. Each *p-*value was compared to its corresponding BH critical value (*i/m*) x *q*, where *i* is the rank order of the *p-*value and *m* is the total number of tests within that family. The largest *p-*value that met the BH criterion was identified, and all *p-*values equal to or smaller than this threshold were considered statistically significant after FDR correction. For primary analyses, three correlation tests were included in each BH correction. For example, (1) correlation between RS-FC in the mirror network and avolition, (2) correlation between RS-FC in the mirror network and anhedonia, and (3) correlation between RS-FC in the mirror network and asociality. Fisher’s R to Z transformations were conducted on significant correlations between RS-FC networks and MAP negative symptom domains that survived correction for multiple comparisons to determine whether the magnitude of the correlation was significantly higher for asociality than the other two MAP domains. The associations between social brain networks and the lower-level EXP negative symptom domains (i.e., blunted affect, alogia) were included in exploratory analyses for completeness rather than hypothesis testing. One-way ANOVAs were conducted to allow comparisons with past studies on RS-FC in PD and EP and determine whether EP and CN show the expected pattern of effects (EP > CN).

### Sensitivity analysis

Since the data had been collected, a sensitivity power analysis was used to determine the minimum detectable effect (MDE), calculated using the sample size, power (1 – β = 0.80), and alpha level (α = 0.05), for each analytic plan separately, to ensure sufficient power for each hypothesis (i.e., hypothesis 1 and 2). Variants of the *R* function pwr were used with a sample size of 114 for bivariate correlations and linear regressions, and 114 EP and 46 CN for ANOVA. For Hypothesis 1, sensitivity analysis with pwr.r.test indicated the study is powered to detect medium correlation effects (critical *r* = −0.23 to 0.23). For Hypothesis 2, pwr.anova revealed the study is powered to detect small to medium effects (*f* = 0.16) for group differences in the social brain networks (*n* = 160, 2 groups).

## Results

### Hypothesis 1: Bivariate correlations

Reduced RS-FC in the mirror network was significantly associated with greater asociality (*r* = −0.20, *p* < 0.05), anhedonia (*r* = −0.23, *p* < 0.05), and avolition (*r* = −0.24, *p* < 0.05; Table 3). Fisher’s R to Z transformations revealed that the association between the mirror network and asociality was not significantly greater than the association with anhedonia (asociality *z* = −0.07, anhedonia = −0.09, *p* = 0.84) or avolition (avolition *z* = −0.08, *p* = 0.94), indicating a lack of specificity for asociality. Reduced RS-FC in the perception network was significantly associated with greater avolition (*r* = −0.19, *p* < 0.05); however, the significance of this correlation did not survive multiple comparison corrections. Exploratory correlations indicated that reduced RS-FC in the mirror (*r* = −0.20, *p* < 0.05) and mentalizing networks (*r* = −0.21, *p* < 0.05) were associated with more severe blunted affect (Table 4).

**Table 3:** Correlations between resting-state functional connectivity and CAINS negative symptom motivation/pleasure domains.

RS-FC Network	Asociality	Anhedonia	Avolition
Affiliation	−0.18	−0.17	−0.14
Aversion	−0.07	−0.06	−0.08
Perception	−0.13	−0.11	−0.19*
Mirror	−0.20*	−0.23*	−0.24*
Mentalizing	−0.17	−0.09	−0.16

Bold indicates that the correlation survived the Benjamini-Hochberg correction for multiple comparisons (FDR = 0.05).

*p < 0.05.

**Table 4:** Exploratory correlations between resting-state functional connectivity and CAINS negative symptom expressivity domains.

RS-FC network	Blunted affect	Alogia
Affiliation	−0.09	−0.19
Aversion	−0.03	−0.15
Perception	−0.10	−0.15
Mirror	−0.20*	−0.14
Mentalizing	−0.21*	−0.13

*p < 0.05.

### Hypothesis 2: Group differences

**Fig. 1:**
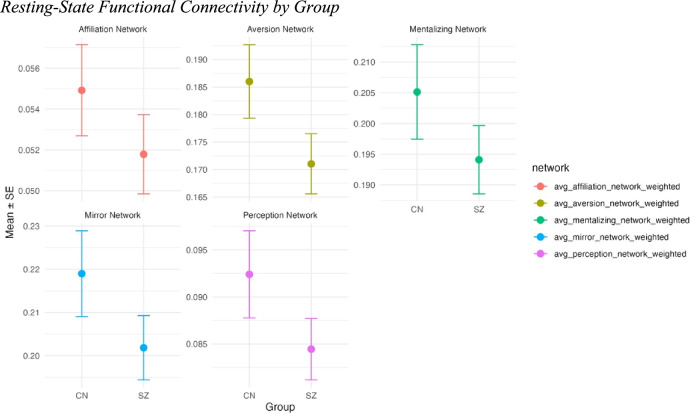
Resting-state functional connectivity by group. Group difference in RS-FC within fine social brain network (EP = 114; CN = 46). EP early psychosis, CN controls. RS-FC resting-state functional connectivity. Error bars represent standard error (SE).

The 2 Group × 5 Network ANOVA revealed a main effect of network (*F* (4,845) = 64.56, *p* < 0.001), indicating that the average RS-FC differed significantly across the five networks. Follow-up analyses via Tukey HSD revealed significant pairwise differences between most networks. The affiliation network had significantly lower RS-FC compared to the other networks (affiliation *M* = 0.05, SD = 0.02, *p* < 0.0001). The aversion network (*M* = 0.19, SD = 0.05) had significantly greater RS-FC than the perception network (*M* = 0.09, SD = 0.04; *p* < 0.0001), but both aversion and perception networks had lower RS-FC than the mirror (*M* = 0.23, SD = 0.07) and mentalizing networks (*M* = 0.21, SD = 0.06; *p* < 0.001). The mirror and mentalizing networks did not significantly differ from each other (*p* = 0.45). The main effect of group was significant such that EP (*M* = 0.06, SD = 0.08) had significantly lower RS-FC compared to CN (*M* = 0.07, SD = 0.08), (*F* (1,845) = 6.64, *p* < 0.01; Fig. 1). There was no significant Network × Group interaction, indicating that the relationship between Network and RS-FC did not differ significantly by group (*F* (4,845) = 0.35, *p* = 0.84).

## Discussion

The current study evaluated the selectivity of the association between RS-FC in brain networks important for social functioning in healthy study samples and negative symptom domains in EP. Hypothesis 2 was supported, but evidence for hypothesis 1 was mixed. Across findings, two main findings emerged. First, the EP group exhibited significantly reduced RS-FC in social brain networks compared to CN, although this effect was not driven by any specific network. This is consistent with past RS-FC studies, indicating EP has reduced RS-FC (i.e., hypoconnectivity) in the DMN compared to CN^36–39^ and other brain networks^35,40–42^. Rutherford et al. found that decreased FC between the left inferior frontal gyrus and intraparietal sulcus in the same HCP-EP dataset was associated with general cognition and global functioning^35^. However, this pattern of hypoconnectivity is not consistent across the literature and may be dependent on factors such as illness phase, antipsychotic use, symptom severity, cognitive impairment, age, education, and specific brain regions involved. Current findings support reduced RS-FC specifically in EP (i.e., within five years of illness onset) compared to CN. Future research should further investigate these factors.

Second, individual negative symptom domains showed differential correlational patterns across the five social brain networks. Since no correlations between negative symptom domains and RS-FC measures demonstrated statistical significance on Fisher’s R to Z, this lends greater support for the two-dimensional conceptualization than the five-factor conceptualization. Past studies have supported both hierarchical and 5-factor structures of negative symptoms^16,45,47,90–94^, emphasizing the need to identify neurobiological correlates of the two broad dimensions (motivation/pleasure and expressivity) along with the five individual domains that comprise them. Current findings are inconsistent with past RS-FC studies showing hyperconnectivity in the DMN but consistent with studies showing hypoconnectivity in the DMN^29–34,36–39^. Current findings are also consistent with research showing DMN hypoconnectivity is associated with worse negative symptom severity in EP^39,50^. The current study contributes to the literature investigating RS-FC in EP before the long-term effects of illness chronicity and antipsychotic usage. Specifically, results indicated that reduced RS-FC in the mirror network is associated with greater asociality, anhedonia, and avolition to a similar extent across these symptoms in EP. The mirror network is involved in interpreting intentions and emotions through sensory and motor processes^69–71^. In EP, dysfunction within mirror network regions is associated with abnormalities in social experience sharing and imitation^71^. Therefore, findings suggest RS-FC within a network implicated in higher-order complex social processes may underlie the motivation/pleasure negative symptom domains. These social brain networks appear to be related to multiple negative symptoms, but not differentially sensitive to asociality as hypothesized. Exploratory analyses indicated that reduced RS-FC in the mirror and mentalizing networks is differentially associated with one of the expressivity domains (i.e., blunted affect). Similar to the mirror network, the mentalizing network is involved in complex social processes such as social cognition and theory of mind. These processes are more closely related to emotional expression and perception than to speech production and fluency, indicating the functional specificity of the mentalizing network for blunted affect over alogia.

Current findings also support past work indicating that individual negative symptom domains that make up the 2 broader dimensions have unique correlations that can be masked when data are examined at the broader dimension level. For example, Cheon et al. and Wang et al. found that more severe avolition is associated with reduced RS-FC and reduced flexibility of functional organization within the DMN and control systems and that these associations are stronger than at the dimension level, emphasizing domain-specific neural mechanisms^45,95^. Ahmed et al. used magnetic resonance spectroscopy (MRS) to examine glutamate and GABA concentrations in the anterior cingulate cortex and found that the MAP dimension was inversely associated with both GABA and glutamate and that this effect was driven by avolition, a domain-specific effect^96^. Paul et al. demonstrated that blunted affect has a stronger association with neurocognition compared to the broader EXP dimension, whereas asociality, avolition, and anhedonia domains relate more to psychosocial functioning compared to the broader MAP dimension^91,92^. The current study found stronger associations between RS-FC within the mirror network for blunted affect compared to alogia, supporting the 5-factor model, although the magnitude of difference between these domains was small.

Current findings are also in part consistent with past studies, indicating that the individual domains travel together at the dimension level. Kaliuzhna et al., Li et al., and Wolpe et al. suggest that finer subdomain models do not significantly outperform broader dimension models, supporting the utility of the two-factor hierarchical model in sufficiently capturing the structure of negative symptoms^97–99^. In the current findings, the mirror network appeared to travel together within the broader motivation/pleasure dimension, which supports the two-dimensional model. Therefore, support exists for the broad dimensions and specific domains capturing unique mechanistic variance, depending on the mechanisms being examined.

Limitations should be considered when interpreting the current results. First, although the study utilized a large neuroimaging dataset, the sample size remains relatively small given the difficulty of recruiting and scanning individuals within the first five years of psychosis onset. A small sample size limits the generalizability of the findings, indicating a need for larger replication studies. Second, the power of the current sample only allowed for the detection of medium effects across both hypotheses. It is possible that smaller domain-specific effects could have gone undetected in the current study. Third, HCP-EP data were specific to the early phase of psychosis and may not extend to chronic psychosis or the prodromal phase. Replication studies are needed across phases of illness. Fourth, given that the sample is a majority of affective psychoses, it is unclear whether the current findings are generalizable to all non-affective psychoses. Future studies should explore mechanistic differences in RS-FC and negative symptoms across diagnostic groups, including primary versus secondary negative symptoms.

Additional research is needed that examines psychological and biological mechanisms of the two dimensions and five domains to identify correlates that are most relevant for treatment at different levels of clinical granularity. If unique domain-specific mechanisms continue to be identified, this may warrant a change in the DSM criteria of negative symptoms. Standard rating scales may benefit from updated scoring practices. Neuroscience methods, such as neuroimaging, may better reflect the underlying pathophysiology of the five negative symptom domains than clinical ratings. These findings inform intervention targets by indicating which social brain circuits are associated with specific dimensions of negative symptoms. For example, the mirror network had a stronger association with the motivational/pleasure (MAP) negative symptom dimension. This circuit could be targeted by neuromodulation techniques, such as repetitive transcranial magnetic stimulation (rTMS), to alter network-level activation. Such protocols have proven effective for improving negative symptoms by targeting other circuits^100^ Specifically targeting social brain circuits may allow for a more personalized focus on the MAP dimension in individuals with that profile of negative symptoms.

## Supplementary information

Supplemental Materials for associations between negative symptoms and resting-state functional connectivity within social brain networks among individuals with early psychosis

## Data Availability

The data that support the findings of this study are available from the corresponding author upon reasonable request.
