# Supplementary material for: Associations between negative symptoms and resting-state functional connectivity within social brain networks among individuals with early psychosis
Source: Schizophrenia (Heidelb). 2026 Apr 17;12(1):51. doi: 10.1038/s41537-026-00756-9 (PMC13273073; doi:10.1038/s41537-026-00756-9)
Supplement: Supplementary file 1 — Supplemental Materials for associations between negative symptoms and resting-state functional connectivity within social brain networks among individuals with early psychosis [file 41537_2026_756_MOESM1_ESM.docx]

**Supplemental Materials for**

**ASSOCIATIONS BETWEEN NEGATIVE SYMPTOMS AND RESTING-STATE FUNCTIONAL CONNECTIVITY WITHIN SOCIAL BRAIN NETWORKS AMONG INDIVIDUALS WITH EARLY PSYCHOSIS**

Anna R. Knippenberg, M.S.

Lawrence H. Sweet, Ph.D.

Lauren Luther, Ph.D.

Somin Kim, M.A.

Gregory P. Strauss, Ph.D.*

Department of Psychology, University of Georgia

Corresponding authors:

*Gregory P. Strauss, Ph.D., Email: gstrauss@uga.edu. Phone: +1-706-542-0307. Fax: +1-706-542-3275. University of Georgia, Department of Psychology, 125 Baldwin St., Athens, GA 30602

| Supplemental Table 1. Descriptive statistics for resting-state functional connectivity in five social brain networks and clinical rating scales in the processed HCP-EP sample and the final analyzed sample (i.e., >9 minutes of analyzable RS-FC data) from EP and CN groups. | | | |
| --- | --- | --- | --- |
|  | Processed Sample | Analyzed Sample | Test statistic, *p* |
|  | CN=55 | CN=46 |  |
|  | *Mean (SD)* | *Mean (SD)* |  |
| *RS-FC Networks* |  |  |  |
| Affiliation | 0.05 (0.02) | 0.05 (0.02) | F(1,99)=0.99, p=0.97 |
| Aversion | 0.19 (0.05) | 0.19 (0.05) | F(1,99)=1.02, p=0.95 |
| Perception | 0.09 (0.03) | 0.09 (0.04) | F(1,99)=0.88, p=0.64 |
| Mirror | 0.22 (0.07) | 0.23 (0.07) | F(1,99)=0.97, p=0.90 |
| Mentalizing | 0.21 (0.06) | 0.21 (0.06) | F(1,99)=0.97, p=0.91 |
|  | EP=117 | EP=114 |  |
|  | *Mean (SD)* | *Mean (SD)* |  |
| *RS-FC Networks* |  |  |  |
| Affiliation | 0.05 (0.02) | 0.05 (0.02) | F(1,229)=0.98, p=0.90 |
| Aversion | 0.17 (0.06) | 0.17 (0.06) | F(1,229)=1.01, p=0.97 |
| Perception | 0.08 (0.04) | 0.08 (0.04) | F(1,229)=0.99, p=0.96 |
| Mirror | 0.20 (0.08) | 0.20 (0.08) | F(1,229)=0.99, p=0.97 |
| Mentalizing | 0.19 (0.06) | 0.19 (0.06) | F(1,229)=0.98, p=0.91 |
| *Clinical Rating Scales* |  |  |  |
| CAINS Asociality | 1.27 (0.91) | 1.25 (0.92) | F(1,229)=0.99, p=0.96 |
| CAINS Anhedonia | 2.07 (0.88) | 2.07 (0.88) | F(1,229)=1.00, p=0.98 |
| CAINS Avolition | 1.43 (0.99) | 1.42 (0.99) | F(1,229)=0.99, p=0.98 |
| CAINS Blunted Affect | 1.10 (1.08) | 1.10 (1.07) | F(1,229)=1.01, p=0.96 |
| CAINS Alogia | 1.12 (0.80) | 1.11 (1.20) | F(1,229)=1.04, p=0.83 |

*Note.* The HCP-EP Release 1.1 included data from 125 EP and 58 CN. 3 EP were missing demographic data, 1 EP had poor alignment, 2 EP had insufficient degrees of freedom, and 2 EP had scanning artifacts. 2 CN had poor alignment, and 1 CN was missing structural MRI data. EP=117 and CN=55 were processed via the rsfMRI processing pipeline. EP = early psychosis. CN = controls. **p* < .05

| Supplemental Table 2. Correlations between CAINS Negative Symptom Domains. | | | | | |
| --- | --- | --- | --- | --- | --- |
|  | Anhedonia | Asociality | Avolition | Blunted Affect | Alogia |
| CAINS Anhedonia | 1.00 | - | - | - | - |
| CAINS Asociality | 0.62*** | 1.00 |  |  |  |
| CAINS Avolition | 0.64*** | 0.62*** | 1.00 |  |  |
| CAINS Blunted Affect | 0.47*** | 0.42*** | 0.44*** | 1.00 |  |
| CAINS Alogia | 0.39*** | 0.28** | 0.30*** | 0.81*** | 1.00 |

*Note.* Pearson’s *r* correlations are reported. CAINS = Clinical Assessment Interview for Negative Symptoms. **p* < .05, ***p* < .01, ******p* < .001.
